# Probiotics in Orthopedics: From Preclinical Studies to Current Applications and Future Perspective

**DOI:** 10.3390/microorganisms11082021

**Published:** 2023-08-06

**Authors:** Antonio Mazzotti, Laura Langone, Alberto Arceri, Elena Artioli, Simone Ottavio Zielli, Simone Bonelli, Pejman Abdi, Cesare Faldini

**Affiliations:** 1 1st Orthopaedics and Traumatologic Clinic, IRCCS Istituto Ortopedico Rizzoli, 40136 Bologna, Italy; antonio.mazzotti@ior.it (A.M.); laura.langone@ior.it (L.L.); elena.artioli@ior.it (E.A.); simoneottavio.zielli@ior.it (S.O.Z.); simone.bonelli@ior.it (S.B.); pejman.abdi@ior.it (P.A.); cesare.faldini@ior.it (C.F.); 2 Department of Biomedical and Neuromotor Sciences (DIBINEM), Alma Mater Studiorum University of Bologna, 40123 Bologna, Italy

**Keywords:** probiotics, orthopedic, bone, osteoporosis, cartilage, muscles, skin, wounds, SAP

## Abstract

In recent years, probiotics have been emerging as an attractive therapeutic strategy for several diseases. In orthopedics, probiotics seem to be a promising supplementation for treatment of osteoporosis, osteoarthritis, muscle loss-related disease, wound and ulcer issues, and prevention of surgical antibiotic prophylaxis side effects. Although probiotics are still not included in guidelines for these conditions, several studies have reported theoretical benefits of their administration. Further high-level clinical trials are necessary to convert research into solid clinical practice. However, probiotics represent a cost-effective future perspective and may play a role in association with traditional orthopedic therapies.

## 1. Introduction

Probiotics were defined in 2013 by an expert panel convened by the International Scientific Association for Probiotics and Prebiotics (ISAPP) as “live microorganisms that, when administered in adequate quantities, confer a health benefit on the host” [1]. This definition includes a broad range of applications that exploit the effects of some probiotics on human health: antimicrobial properties, inhibition of pathogen adhesion and cellular invasion, occupation of space otherwise occupied by pathogens, and growth limitation by bacterial competition [2].

The most studied species include *Bifidobacterium*, *Saccharomyces*, and *Lactobacillus* (as defined before the taxonomy’s reorganisation in 2020) [3]. High-quality evidence studies show the efficacy of probiotic administration for gastrointestinal disorders, particularly for the treatment of acute infectious diarrhoea, antibiotic-associated diarrhoea, *Clostridium difficile*-associated diarrhoea, hepatic encephalopathy, ulcerative colitis, irritable bowel syndrome, functional gastrointestinal disorders, and necrotising enterocolitis [4].

The effect of probiotics can also be extended to other areas: in neurology for the prevention and treatment of neurodegenerative and demyelinating diseases [5,6]; in pain control, as probiotic supplementation appears to increase pain thresholds [7]; in psychiatry, for the treatment of stress- and depression-related behaviours [8] and obsessive-compulsive behaviours [9]; in dermatology, for the treatment of neurogenic skin inflammation, acne vulgaris, acne rosacea [10,11], psoriasis and atopic dermatitis [12], and aging skin [13]. Probiotics may also be used in orthopedics for the treatment of various pathological conditions. This narrative review aims to investigate the literature regarding preclinical studies, current applications, and future perspectives for probiotics in orthopedics. In particular, the following topics will be analysed: bone and cartilage defects, muscle diseases, chronic wounds, and surgical antibiotic prophylaxis.

## 2. Bone

Bone is a specialised connective tissue that consists of the calcified extracellular substance, the bone matrix, and the cell component (osteocytes, osteoblasts, and osteoclasts).

Bone tissue is always in the balance of bone formation and resorption [14]. This mechanism, so-called bone remodeling, is responsible for the formation and maintenance of bone functionality, and it is regulated by different stimuli. Bone remodeling is able to replace around 5–10% of existing bone every year [15]. Osteoclasts and osteoblasts form the so-called bone remodeling unit (BRU). Osteoblasts produce the organic bone matrix and facilitate bone mineralisation, whereas osteoclasts are responsible for the degradation of bone and extracellular matrix. Dysregulation in ion numbers and the imbalance between osteoclast and osteoblast action can lead to bone disorders, such as osteoporosis, Paget, osteogenesis imperfecta, rickets, ostheomalacia, renal osteodystrophy, and hyperparathyroidism [16]. Osteoporosis is by far the most frequent bone metabolic disorder, affecting nearly 22% of women and 7% of men (older than 50) in 27 European countries, as reported by the World Health Organization (WHO) [17]. Risk factors include non-modifiable intrinsic factors, such as genetic factors and aging, and extrinsic modifiable factors, such as drugs. Drugs that could interfere with bone health include glucocorticoids, aromatase inhibitors (i.e., anastrozole and letrozole), medroxyprogesterone acetate, thiazolidinediones, proton pump inhibitors (PPIs) and antiepileptics, heparin, and serotonin-selective reuptake inhibitors [18].

In addition, the gastrointestinal system plays a crucial role in maintaining bone health by absorbing calcium, phosphorus, and magnesium, which are fundamental elements for bone mineralisation, and by producing endocrine factors such as incretin and serotonin, which are signal molecules that can interact with receptors of bone cells. For this reason, the gut microbiota has been proposed as a regulator of bone health [19].

The main treatment for osteoporosis is pharmacological, including bisphosphonates (such as alendronate or ibandronate), hormone therapy (estrogens), calcitonin, denosumab (RANKL/RANK inhibitor), parathyroid hormone (PTH), and analogs. Nevertheless, the implication of the microbiota as a regulator of bone health may suggest a possible use of probiotics in the treatment and prevention of bone disease [20].

Several preclinical studies have been conducted to analyse the probiotic pathway of action on bone health, focusing on the RANKL/RANK/OPG pathway and histone methylations [21,22]. Amin et al. [21] investigated the RANKL/RANK/OPG pathway, responsible for regulating osteoclast activity, but found no evidence of probiotic efficacy through this pathway. Behera et al. [22] reported promising results evaluating the effects of probiotic supplementation on mitochondrial biogenesis and bone homeostasis through the histone methylation mechanism in obese mouse models. Osteoporosis may be related to attenuation of osteoblast differentiation through hypermethylation of the H3K27me3 mark at the Tfam promoter. Inhibition of Tfam due to hypermethylation exacerbated glycolysis rate and mitochondrial bioenergetics metabolism and subsequently inhibited osteogenesis and bone formation, causing obesity-induced metabolic osteoporosis. The study reported that probiotic treatment increased mitochondrial Tfam expression in osteoblasts and promoted Kdm6b/Jmjd3 histone demethylase, which inhibits H3K27me3 epigenetic methylation at the Tfam promoter.

Concerning animal studies, in a 2021 review, Malmir et al. [23] found 37 animal studies analysing probiotic effects on bone. The main probiotics administered were *L. reuteri* (ATCC PTA 6475), *L. casei* (KFRI-127), *L. paracasei* (NTU 101, NTU 102, HII01), *L. plantarum* (DSM 15312, DSM 15313, NK3), *L. acidophilus* (ATCC 4356), *B. bifidum* (NCIM 5697), *B. longum* (NCIM 5672, NK49), *B. subtilis* (C-3102), *L. helveticus* (LBK-16H, ATCC 27558), *L. bulgaricus*, *Entrococcos faecium*, *L. rhamnosus*, *B. breve* (NCIM 5671), *B. animalis*, *Streptococcus thermophilus*, *Pediococcus acidilactici*, *Escherichia coli*, *Lactococcus lactis* (H61, G50), *Bacillus licheniformis*, *Clostridium butyrium*, *Bacillus coagulans*, and *Pasteurized Akkermansia muciniphila.* Some studies in this review reported an increase in calcium, phosphorus, 25-OH-D, PTH, osteocalcin (OC), and alkaline phosphatase (ALP) levels after probiotic feeding, while others reported a decrease in ALP, acid phosphatase (ACP), and urinary calcium and phosphorus levels. Improvements in bone mass density and bone mineral content were reported after probiotic supplementation in most studies but only in eight trials of the examined review study.

Clinical trials seem to confirm what was found in preclinical and animal studies. However, the bacteria varied between trials and probably worked through different or overlapping mechanisms.

*L. reuteri* 6475 acts by suppressing the gene expression of pro-inflammatory and pro-osteoclastogenic cytokines, both in the gut and bone marrow [24,25,26], as well as lactobacilli such as LGG and VSL#3 [27]. The anti-inflammatory effect on the gut may improve calcium transport across the intestinal barrier, while bifidobacteria produce short-chain fatty acids (SCFAs) that can lower the pH of the intestinal tract and subsequently increase mineral absorption [28].

*L. helveticus* LBK-16H acts via two mechanisms to improve bone mineral density (BMD): by increasing calcium absorption and by producing the bioactive forms of isoleucyl-prolyl-proline (IPP) and valyl-prolyl-proline (VPP), two peptides capable of inhibiting angiotensin-converting enzyme (ACE), thereby preventing the formation of angiotensin II (Ang II), a stimulator of OC resorption [29]. Always with BMD in mind, Takimoto et al. [30] studied the effects of *Bacillus subtilis* C-3102 (C-3102) in seventy-six healthy postmenopausal Japanese women for 24 weeks. Compared to placebo, C-3102 significantly increased total hip BMD (placebo = 0.83 ± 0.63%, C-3102 = 2.53 ± 0.52%, *p* = 0.043). In addition, the effect on gut microbiota was analysed and showed a significant increase in Bifidobacterium and a significant decrease in Fusobacterium in the C-3102 group at 12 weeks compared to baseline. It is likely that C-3102 improves BMD by inhibiting bone resorption and modulating the gut microbiota in healthy postmenopausal women.

However, after administration of probiotics to the gut, such as *Lactobacillus acidophilus* DDS-1, *Bifidobacterium lactis* UABla-12, *Bifidobacterium longum* UABl-14, and *Bifidobacterium bifidum* UABb-10, and with the abundant presence of gut bacteria associated with beneficial health effects, especially *Bifidobacterium* and *Lactobacillus*, there is not always correlation with a change in terms of bone mineral content [31].

Regardless of the action mechanism, some high-evidence studies showed promising metabolic effects. Narva et al. [29] studied the effect of milk fermented with *L. helveticus* on acute changes in calcium metabolism in postmenopausal women, reporting a reduction in serum parathyroid hormone (PTH) and an increase in serum calcium. Again, Jones et al. [32], in a double-blind, placebo-controlled, randomised, parallel-arm, multicenter study, investigated the changes in serum low-density lipoprotein cholesterol over a 9-week administration of *Lactobacillus reuteri* NCIMB 30242. Authors reported an increase in circulating 25-hydroxyvitamin D in response to oral probiotic supplementation, suggesting a possible way for the prevention of low serum 25-hydroxyvitamin D-related osteoporosis [32]. Also, Nilsson et al. [33] reported a reduction in loss of total BMD in patients treated daily with *L. reuteri* 6475 in a double-blind, placebo-controlled study. Jafarnejad et al. [34], in a randomised, double-blind, placebo-controlled clinical trial, supplemented postmenopausal women patients with GeriLact containing seven probiotic bacterial species, plus 500 mg calcium and 200 IU vitamin D daily for 6 months. The multi-species probiotic significantly reduced bone-specific alkaline phosphatase (*p* = 0.03), collagen type 1 cross-linked C-telopeptide (*p* = 0.04), and serum PTH (*p* = 0.01) and TNF-α (*p* = 0.02) in the intervention group compared with the placebo group but had no effect on spine and total hip BMD.

Some studies have suggested the addition of probiotics to traditional drugs for osteoporosis treatment. Jia et al. [35] conducted a randomised, placebo-controlled clinical trial in which zoledronic acid and calcitriol were administered together with bifidobacteria quadruple viable tablets and reported an improvement in bone metabolism and intestinal flora in patients with osteoporosis. Similarly, Lambert et al. [36] administered a red clover extract rich in isoflavone aglycones and probiotics to simultaneously promote absorption and a favourable intestinal bacterial profile to enhance isoflavone bioavailability for the treatment of postmenopausal osteopenic women supplemented with calcium (1200 mg/d), magnesium (550 mg/d), and calcitriol (25 μg/d). This study showed that one year of red clover extract supplementation effectively attenuated estrogen deficiency-induced BMD loss, improved bone turnover, promoted a favourable estrogen metabolite profile (2-OH:16α-OH), and stimulated equol production.

In summary, clinical and animal studies have shown promising effects of probiotics on bone with varying mechanisms of action. Probiotics have been mainly associated with improvements in bone mineral density and metabolic effects. Some studies also suggest combining probiotics with traditional osteoporosis drugs for better outcomes [23,29,35,36] (Table 1).

## 3. Cartilage

Cartilage is a type of connective tissue with highly specialised cells called chondrocytes, which are embedded in a matrix of collagens, proteoglycans, and non-collagenous proteins that protect the cells from normal use. The cartilage of the joints is called articular cartilage, which allows low-friction movement of the synovial joints [37]. During life, articular cartilage undergoes internal remodeling as cells replace matrix macromolecules lost to degradation. With age, the ability of chondrocytes to maintain and rebuild articular cartilage decreases, increasing the risk of articular cartilage surface degeneration. Progressive degeneration of articular cartilage, also known as osteoarthritis (OA), is characterised by joint pain and dysfunction [38].

OA is the most common disabling disease in older people [39]. Due to their beneficial properties, probiotics may represent a valid adjunct to traditional OA treatment. Despite this, no human studies have been reported investigating the effect of probiotics on OA. To date, only preclinical in vitro and in vivo studies have been conducted, with promising and encouraging results (Table 2).

Korotkyi et al. [40] investigated the chondroprotective effect of probiotics in OA-induced knee joints in rats. The study analysed the use of chondroprotectants and probiotics separately and in parallel. Separate use of chondroprotectants and probiotics had a positive effect on preventing cartilage destruction. Therefore, the use of a chondroprotectant enhances the positive effect of probiotic microbiota in anti-inflammatory processes [40].

A study tried to evaluate the role of the microbiome in the pathogenesis of OA [41]. The authors performed antibiotic-induced ablation of the microbiome followed by reconstitution and administration of probiotics in a mouse model of 21 male C57BL/6 mice with OA induced. Then, a mixture of probiotic strains *Lacticaseibacillus paracasei* 8700:2 (DSM13434), *Lactiplantibacillus plantarum* HEAL9 (DSM 15312), and *Lactiplantibacillus plantarum* HEAL19 (DSM 12313) in equal amounts (*n*  =  11), or vehicle (glycerol) (*n*  =  10) was administered for 10 weeks. Knee joints were scanned by MicroCT for quantitative and qualitative changes in subchondral bone, followed by histological examination of cartilage to quantify severity. Osteoarthritis Research Society International (OARSI) scores at the medial femoral condyle (MFC) were significantly lower in the faecal microbial transplant and probiotic-treated mice compared to the control group (4.64 ± 0.32 compared to 6.48 ± 0.53). There were no significant differences in inflammation scores and circulating inflammatory cytokines between the two groups [41]. This finding could suggest that the anti-inflammatory probiotic effects may differ relative to tissue type, such as bone or cartilage. Thus, the action mechanism should be investigated to understand the pathway of the probiotic chondroprotective effects.

Although no human studies have been reported, preclinical studies in rats and mice demonstrated the chondroprotective effects of probiotics and their role in reducing cartilage destruction and improving OA severity.

## 4. Muscles

Skeletal muscle represents 40% of the total mass of the human body and can be considered a reservoir of protein to be used in catabolic situations. Daily cycles of dietary anabolism/catabolism can be modified in the long term by age, physiological status, lifestyle and fitness, resulting in muscle mass gain (recovery after exercise and endurance) or loss (sarcopenia and cachexia) [42].

Microbiota may interfere with the regulation of skeletal muscle responsiveness to anabolic stimuli. For this reason, protein supplementation and micronutrients are used to improve or limit the loss of muscle mass. Recently, scientific research has included pro-biotics as a supplement to target muscle mass and function [43].

For a long time, marketed probiotics were mainly lactic acid bacteria (LAB) and bifidobacterial strains of human or food origin, but current strategies focus more on using bacteria of human origin [44].

Applications focus on both muscle gain and muscle loss, studying athletes or cachectic and sarcopenic patients (Table 3).

### 4.1. Muscle Mass Gain

In 2019, the International Society of Sports Nutrition (ISSN) published an objective and critical review of the mechanisms and use of probiotic supplementation in improving the health, performance, and recovery of athletes based on the currently available literature, reporting the following sentence as a conclusion: “The administration of selected anti-inflammatory probiotic strains has been associated with improved recovery from muscle-damaging exercise” [54]. In fact, probiotic supplementation may have the potential to remove and utilise blood lactate produced after exercise. Hsu et al. [45] observed that consuming a kefir LAB strain containing *Lactobacillus fermentum* DSM 32784 (LF26), *L. helveticus* DSM 32787 (LH43), *L. paracasei* DSM 32785 (LPC12), *L. rhamnosus* DSM 32786 (LRH10), and *Streptococcus thermophilus* DSM 32788 (ST30), every day over 4 weeks, swimming time-to-exhaustion was significantly longer, forelimb grip strength was higher, and serum lactate, ammonia, blood urea nitrogen (BUN), and creatine kinase levels were lower after the swimming test.

Moreover, probiotic supplementation could also have benefits for an athlete’s body composition, decreasing levels of fat mass and increasing fat-free mass [46,55,56]. Nowadays, this is only demonstrated in animal models. Chen et al. [47] orally administered *L. plantarum* TWK10 (LP10) to mice for six weeks and examined the relative muscle weight, measured by combining the gastrocnemius and soleus muscles. This study observed that the relative muscle weight was significantly increased, with a marked gain of the type I muscle fibre number.

A recent systematic review and meta-analysis of clinical trials [48] investigated the effect of probiotic supplementation on muscle mass, total lean mass, and muscle strength in both young and older adults. The study reported that muscle mass was improved by probiotics compared to placebo (SMD: 0.42, 95% CI: 0.10–0.74, I^2^ = 57%, *p* = 0.009), but no changes were demonstrated in relation to total fat-free mass (k = 12; SMD: −0.03, 95% CI: −0.19–0.13, I^2^ = 0%, *p* = 0.69). A significant increase in global muscle strength was also observed (SMD: 0.69, 95% CI: 0.33–1.06, I^2^ = 64%, *p* = 0.0002).

Conversely, another systemic review [49] found no evidence to support the hypothesis that probiotics can improve endurance and aerobic exercise performance, but the analysis focused on cardiorespiratory fitness markers without considering muscle mass and strength.

### 4.2. Prevention of Muscle Mass Loss (Sarcopenia and Cachexia)

Sarcopenia is a progressive skeletal muscle disease associated with an involuntary accelerated loss of muscle mass and an increase in adverse events such as falls, functional decline, frailty, and mortality, especially in older adults [57] and sometimes in younger patients [58]. The prognosis depends on several factors, including physical inactivity, hormonal imbalances, sleep disturbances, and malnutrition [59,60,61,62]. Although this topic may seem distant from the orthopedic field, it is becoming increasingly important, as it has been demonstrated to impact the outcomes of major orthopedic surgeries such as hip arthroplasty and spinal surgery [63].

A multidisciplinary approach that includes orthopedic consideration of sarcopenia and any treatment that may be able to reduce it can, therefore, be crucial in enhancing postoperative recovery for patients.

Sarcopenic patients are susceptible to loss of strength due to muscle fibre loss caused by overproduction of reactive oxygen species and pro-inflammatory mediators, immune senescence, and anabolic resistance status [64,65,66,67]. The main treatments for sarcopenia are nutritional therapies aimed at achieving a caloric intake of 24–36 kcal/kg body weight/day and a protein intake of 1.0–1.5 g/kg body weight/day [68,69] and supplementation with antioxidants [70], protein and essential amino acids (EAA) [71], omega-3 fatty acids, and creatine monohydrate [72].

Probiotics have been proposed as a potential nutritional supplement in sarcopenia therapy. In vivo studies in mice supplemented with Lactobacillus showed an increase in muscle mass preservation, a reduction in low-grade inflammation, and an increase in mitochondrial function [47,50,51,52,73]. Fielding et al. [73] reported an increase in muscle strength but not muscle mass.

Cachexia, which is partly related to sarcopenia, is also a muscle wasting disease caused by a multifactorial disorder and characterised by generalised fatigue, loss of body weight, skeletal muscle and fat mass, and reduced food intake. Cachexia is often associated with cancer [74,75] and a systemic inflammatory state, with high pro-inflammatory markers such as C-reactive protein (CRP) [76].

An altered microbiota was obserrved in cancer cachexia, associated with a decrease in *Bifidobacterium*, *Lactobacillus*, and *Faecalibacterium genera* and an increase in *Enterobacteriaceae* and *Enterococcus* [77,78].

LAB were tested in two studies using cancer-induced cachexia rodents. Bindels et al. administered *Limosilactobacillus reuteri* 100–23 and *Lactobacillus gasseri* 311,476 to leukemic mice for 2 weeks. They found a decrease in the levels of markers associated with muscle atrophy and in the release of systemic inflammatory cytokines. Nonetheless, it was hypothesised that the observed beneficial effects might be specific to certain species because the same study did not show these effects for *Lactobacillus acidophilus* [52]. In another study, Varian et al. administered *L. reuteri* ATCC-PTA-6475 for 3 months to mice affected by colorectal cancer. The study revealed a rise in muscle mass and a decline in muscle atrophy. Additionally, there was a decrease in the activity of a gene linked to systemic inflammation [53].

The microbiota has been found to influence skeletal muscle responsiveness to anabolic stimuli, and probiotic supplementation has been explored to improve muscle mass and function. Studies have shown that several specific probiotic strains can aid in muscle recovery after exercise and enhance endurance. However, the evidence is limited. Probiotics have shown potential in the treatment of conditions like sarcopenia and cachexia. Animal studies have demonstrated that probiotic supplementation can preserve muscle mass, reduce inflammation, and enhance mitochondrial function. However, the effects on muscle strength and muscle mass preservation may vary.

## 5. Skin

The skin on the surface is colonised by bacteria, fungi, viruses, micro-eukaryotes (mites), archaea, and sweat, sebaceous glands, and associated hair follicle phages [79]. The microbiome composition depends on several factors, including age, gender, genetics, immunity, hormonal balance, sleep routine, stress, metabolic factors, hygiene and skin care routine, chemical or ultraviolet radiation exposure, physical activity, climate, environmental pollution, and nutrient availability [80]. Most of the skin microbiome is made of bacteria; of these, *Staphylococcus epidermidis*, *Cutibacterium acnes*, and *Corynebacterium* are the most represented [81].

Antibiotic therapies, whose side effect could be the dysbiosis of the gut microbiome’s composition [82], could probably also interfere in the skin microbiome by reason of an interaction between the gut microbiome and the skin microbiome, which is actually not fully understood [83].

Probiotics modifying the gut microbiome may be used for targeting orthopedic skin-related problems, such as chronic wounds [84,85,86] and diabetic foot ulcers (DFU).

Chronic wounds are defined as a pathological condition with a lack of clinical improvement within four weeks from the beginning of treatment and the absence of healing after a period of three months [87]. Microbial infections are among the main causes of delayed wound healing [88]. An exposed epithelial barrier with devitalised tissue, a moist nutrient-rich environment, and dysregulated inflammatory processes are all conditions that create a favourable environment for microbial proliferation [88]. The first microbial contamination is reversible, but after a while a biofilm is produced, conferring functional resistance to antibiotics and the immune system [87]. It is reported that around 60–90% of chronic wounds contain biofilm-forming bacteria [88], and the most common isolated bacteria are *Staphylococcus aureus*, *Pseudomonas aeruginosa* and *β-hemolytic Streptococci*, *Enterococcus* spp., *Klebsiella pneumoniae*, *Acinetobacter baumanii* and *Enterobacter* spp. (ESKAPE pathogens), coagulase-negative *Staphylococci*, and *Proteus* spp.

In chronic wounds, a reduction in migratory and proliferative capability of keratinocytes and fibroblasts was observed. The presence of those bacteria seems to stimulate the influx of immune cells, such as neutrophils and macrophages, but with reduced chemotactic, antimicrobial, and phagocytic activities [88]. This leads to biofilm growth and dysfunctional immune cell infiltration, resulting in a pro-inflammatory state due to excessive toll-like receptor signaling, with a massive release of cytokines, chemokines, and growth factors [89].

Conventional treatment of chronic wounds is based on the use of antimicrobials and antibiotics [90], consisting of local antiseptics and topical and systemic antibiotics. Nevertheless, the bacterial biofilm makes chronic wounds become highly resistant to antimicrobials and antibiotics [88].

The topic of probiotics use seems to be an emerging and interesting option in chronically infected wound management [91]. Topical probiotics penetrate the intercellular lipid matrix, and when they reach the dermis, they activate toll-like receptors. Toll-like receptors are type 1 transmembrane proteins that act as a major signaling receptor for pathogen-associated molecular patterns. Toll-like receptors upregulate collagen and elastin and improve skin clarity, texture, and appearance. Then, probiotic bioactivities throughout toll-like receptors lead to beta-defensin production, which raises the skin’s immune functions [88,92,93]. This might lead to the healing of diabetic ulcers and to the prevention of diabetic foot infections.

In a randomised, double-blind, placebo-controlled trial [92], a series of 60 patients (aged 40–85 years old) with grade 3 DFU were administered *L. acidophilus*, *L. Casei*, *L. fermentum*, and *B. bifidum* for 12 weeks. Significant improvements were seen in DFU length (−1.3 ± 0.9 vs. −0.8 ± 0.7 cm, *p* = 0.01), width (−1.1 ± 0.7 vs. −0.7 ± 0.7 cm, *p* = 0.02), and depth (−0.5 ± 0.3 vs. −0.3 ± 0.3 cm, *p* = 0.02). Improvements in glycaemic control, total cholesterol, high-sensitivity C-reactive protein, plasma nitric oxide, total antioxidant capacity, and malondialdehyde levels were also reported.

In a prospective uncontrolled study [93], *L. plantarum* was administered to 14 patients with diabetes and 20 patients without diabetes for chronic venous leg ulcers. After 30 days of follow-up, ulcer size was reduced by 90% in 43% of the diabetic patients and 50% of the non-diabetic patients. These results may suggest that probiotics break down biofilm, control IL-8 levels produced by polymorphonuclear leukocytes, and regulate the immune system.

In a study [94], the outcomes of burn healing in eight burned patients treated with *L. plantarum* were compared to sulphadiazine treatment for 10 days of daily treatment. In delayed second-degree burns, the administration of *L. plantarum* was as effective as the SD-Ag relative to the decrease in bacterial load, the promotion of granulation tissue, and wound healing. In delayed third-degree burns, results suggested a better outcome for *L. plantarum* treatment [94].

In summary, probiotics can be used topically to improve chronic wound healing and DFU through the effects of the immune function’s activation, bacterial load reduction, and collagen production promotion. Although probiotics are primarily used in chronic wounds, their ability to modulate the host’s immune response and exhibit antimicrobial activity could potentially extend their role in enhancing wound healing even in acute settings, such as acute post-traumatic and post-surgical wounds (Table 4).

## 6. Prevention of Side Effects of Surgical Antibiotic Prophylaxis

In orthopedics, as in surgery in general, surgical site infections (SSIs) are among the most common healthcare-associated infections (HAIs), with a prevalence of 31.0% of all HAIs in hospitalised patients [95]. Guidelines from the Centers for Disease Control and Prevention (CDC) recommend surgical antibiotic prophylaxis (SAP) to reduce the risk of SSIs [96].

However, SAP has some side effects, such as allergy, anaphylaxis, nausea, emergence of antibiotic resistance, and antibiotic-associated diarrhoea due to *Clostridioides difficile* infection (CDI). The incidence of CDI is high in elderly orthopedic patients and has increased significantly in Europe, North America, and Asia since 2000 [97].

Co-administration of probiotics and SAP may be a potential strategy to prevent both CDI and *Clostridium difficile*-associated diarrhoea (CDAD) in orthopedic patients. Probiotics have been shown to produce bacteriocins and defensins, compete with pathogenic bacteria for resources, interfere with bacterial attachment or translocation, reduce luminal pH, and improve intestinal barrier function by increasing mucus production [98].

Two systematic reviews with meta-regression analysis showed that administering probiotics closer to the first dose of antibiotics reduced the risk of CDI by more than 50% in hospitalised adult patients [99] and reported a significant reduction in the rate of developing CDAD in patients receiving antibiotics [100].

Nagamine et al. [101] reported a CDI incidence reduction (95% CI: 0.010–0.565; *p* = 0.002) in elderly orthopedic patients treated with a combination of antibiotics and probiotics, including *Streptococcus faecalis* 2 × 10^8^ CFU/day, *Bacillus mesentericus* 1 × 10^7^ CFU/day, and *Clostridium butyricum* 5 × 10^7^ CFU/day (Bio-Three tablets^®^, Toa Pharmaceutical Co., Ltd., Tokyo, Japan).

In a randomised controlled study, Kaku et al. [102] tried to explain the probiotic effect against SAP side effects, showing that SAP did not influence the entire gut microbiome composition. The authors observed a relative abundance of *S. gallolyticus* after SAP, while probiotics administration significantly reduced the relative abundance of *S. gallolyticus*. Considering the pathogenicity of *S. gallolyticus* and the relationship with SAP, it could be supposed that some SAP side effects are linked to *S. gallolyticus*; thus, probiotics may prevent SAP side effects after surgery (Table 5).

## 7. Discussion

In recent years, probiotics have emerged as an attractive therapeutic strategy in modern medicine for several diseases. Although probiotics are not yet included in guidelines for the treatment of orthopedic pathologies, several studies have been conducted to evaluate the possible benefits of their administration. In orthopedics, probiotics have potential applications in bone, cartilage and muscle diseases, wounds and ulcers, or SAP side effects.

The probiotic effect is expressed at the level of the gut microbiota, which is considered to be an organ capable of regulating bone metabolism through modulation of the immune system, endocrine organs, and calcium activity [2].

Osteoporosis is a widespread disease that may benefit from this treatment; in various studies, probiotics have shown an improvement in bone mineral density and metabolic effects without side effects [20]. For this reason, probiotics could be a safe and effective alternative for preventing bone loss, possibly in combination with traditional therapies [19].

However, most of the papers in the literature were preclinical in vitro and in vivo studies. Few clinical trials in the literature evaluated probiotic effects, and most did not use randomisation or blinding, had participants with different health statuses and comorbidities, and used different probiotic species and doses.

Another common disease is OA, the conservative treatment of which is hotly debated. The use of probiotics in the treatment of OA has been reported, both as a single dose and in combination with chondroprotective drugs [38,39]. However, no human studies have been reported in the literature that investigate the effect of probiotics on OA. To date, only preclinical in vitro and in vivo studies have been conducted, with promising results. Further human studies should be conducted to confirm the therapeutic potential of this intervention in the treatment of OA.

Probiotic supplementation may be beneficial for body composition and strength, particularly for improving muscle mass gain and preventing muscle mass loss in sarcopenic and cachectic patients [31]. The effect of probiotics on muscles may be important in terms of functional recovery and in the possibility of early rehabilitation in post-traumatic and post-operative patients, especially the elderly. The use of probiotics limited the loss of skeletal muscle mass in in vivo animal studies. Again, human studies should be carried out to confirm these results in order to include probiotic administration in treatment guidelines for sarcopenia and cachexia [53,77,78].

Probiotics have shown beneficial effects on diabetic ulcers and chronic wounds, particularly against some types of pathogens. Preliminary evidence supports the use of specific strains in the management of infected chronic wounds [82]. However, it is important to consider the possibility of allergic reactions and local infections due to probiotics becoming pathogens, especially in immunocompromised patients [82]. Incidentally, there is a lack of reported adverse events in most probiotic clinical trials.

Finally, many high-quality studies have shown that probiotic supplementation may be a cost-effective strategy for preventing CDI in hospitalised adults receiving antibiotics [90,91,92,94].

Probiotics cannot replace conventional orthopedic therapy. Current human studies looking at the effects of probiotics are small and show limited evidence. This is probably due to the difficulty in designing an appropriate trial, including ethical issues.

Probiotics are cost-effective, and all studies, although not always achieving statistical significance, reported promising results after probiotic supplementation in major orthopedic conditions, suggesting that probiotics may play a conventional role in the future in conjunction with traditional therapies. Among these, bone infections could also be included; however, currently, there are no clinical studies on this matter, but only a few data derived from animal studies [103].

Exploring modulation of the gut microbiota, regulation of the immune system, and other relevant pathways would be an intriguing avenue to pursue. Additionally, investigating the impact of probiotic bacteria on receptors in human cells and their interaction with other organ systems—especially in terms of whether these effects are direct or mediated by changes in the overall composition of the microbiota following probiotic intervention—is significant for understanding the mechanisms described in this paper.

## 8. Conclusions

Probiotics appear to be a readily available, cost-effective, and promising adjunct for the treatment of osteoporosis, OA, muscle wasting disorders, wound and ulcer problems, and the prevention of SAP side effects. Modulation of the microbiota by probiotics represents a future perspective for the development of routine nutritional or pharmaceutical tools. However, further high-level clinical trials are needed to translate research into clinical practice and to refine the clinical indication of specific probiotic strains.

## Figures and Tables

**Table 1:** Summary of the reviewed studies concerning bone.

Study	Study Population	Probiotic Strain	Duration of the Treatment	Results
Narva et al. [29] Randomised double-blind crossover study	20 postmenopausal women (mean age 65, range 50–78)	*L. helveticus-*fermented milk and *L. helveticus-*derived peptides	2 study days and 6 days washout between each study day	*L. helveticus*-fermented milk reduced serum PTH and increased serum calcium. *L. helveticus*-derived peptides had no significant acute effect.
Jones et al. [32] Double-blind, placebo-controlled, randomised, parallel-arm multicenter study	127 healthy hypercholesterolemic adults (ages 20–75)	*L. reuteri* capsules	13 weeks	Serum 25-hydroxyvitamin D increased by 25.5%.
Nilsson et al. [33] Double-blind placebo-controlled study	70 women with low bone mineral density	10^10^ colony-forming units of *L. reuteri* 6475	12 months	*L. reuteri* 6475 reduced loss of total bone mineral density compared to placebo.
Jafarnejad et al. [34] Randomised, double-blind, placebo-controlled clinical trial	50 women (ages 50–72) with mild bone loss	Multispecies probiotic capsules (GeriLact)	6 months	Decrease in bone-specific alkaline phosphatase and in collagen type 1 cross-linked C-telopeptide in serum PTH and TNF-alfa.
Takimoto et al. [30]	76 healthy, postmenopausal women (50–69 years)	*Bacillus subtilis* C-3102 (C-3102)	24 weeks	Significant increase in total hip bone mineral density. Significant decrease in bone resorption markers.
Jia et al. [35] Placebo-controlled intervention clinical trial	126 elderly hospitalised patients with primary osteoporosis	Bifidobacterium quadruple viable that comprises four components of bifidobacterium, *Lactobacillus acidophilus*, *Enterococcus faecalis*, and *Bacillus cereus*	24 months	Decrease in bone Gla protein, total propeptide of type I procollagen, and *β*-crosslaps. Decrease in phosphate, IL-6 and TNF-*α* serum levels. Increase in IGF-1.
Lambert et al. [36] Parallel-design, placebo-controlled, double-blind, randomised controlled trial	85 postmenopausal women	Heterogeneous culture of probiotic lactic acid bacteria	12 months	Attenuation of bone mineral density loss. Decrease in plasma concentrations of collagen type 1 cross-linked C-telopeptide. No significant effect on other bone turnover biomarkers.

**Table 2:** Summary of the reviewed studies concerning cartilage.

Study	Study Population	Probiotic Strain	Duration of the Treatment	Results
Korotkyi et al. [40] Double-controlled intervention clinical trial	90 white male Wistar rats	Chondroprotector and the probiotic separately and alongside	30 days	Separate chondroprotector and probiotic application seems to prevent cartilage destruction.
Sophocleus et al. [41] Placebo-controlled clinical trial	21 male C57BL/6 mice underwent antibiotic-induced ablation of the microbiome and osteoarthritis induced	*Lacticaseibacillus paracasei* 8700:2 (DSM13434), *Lactiplantibacillus plantarum* HEAL9 (DSM 15,312), and *Lactiplantibacillus plantarum* HEAL19 (DSM 12,313) in equal amounts (*n* = 11)	10 weeks	Inhibition of DMM-induced cartilage damage and impacts on the structure of subchondral bone.

**Table 3:** Summary of the reviewed studies concerning muscles.

Study	Study Population	Probiotic Strain	Duration of the Treatment	Results
Hsu et al. [45]	32 male mice	*Lactobacillus fermentum* DSM 32784 (LF26), *L. helveticus* DSM 32787 (LH43), *L. paracasei* DSM 32785 (LPC12), *L. rhamnosus* DSM 32786 (LRH10), and *Streptococcus thermophilus* DSM 32788 (ST30)	4 weeks	Supplementation alters the gut microbiota composition, improves performance, and combats physical fatigue.
Toohey et al. [46]	23 female athletes (19.6 ± 1.0 years, 67.5 ± 7.4 kg, and 170.6 ± 6.8 cm)	*Bacillus subtilis*	10 weeks	No effect on physical performance but may improve body composition.
Chen et al. [47]	24 mice	*L. plantarum* TWK10 (LP10)	6 weeks	LP10 significantly decreased final body weight and increased relative muscle weight, strength, and endurance. Moreover, a decrease in lactate, ammonia, creatine kinase, and glucose serum levels after acute exercise challenge was observed.
Prokopidis et al. [48] Systematic review and meta-analysis	/	/	/	Probiotic supplementation enhances muscle mass and strength; no effects on total lean mass.
De Pavia et al. [49] Systematic review	/	/	/	Not enough evidence to support that probiotics can improve performance in endurance and aerobic exercises.
Chen et al. [50]	18 female senescence-accelerated mice	*Lactobacillus paracasei* PS23 (LPPS23)	12 weeks	Significant attenuation of age-related decrease in muscle mass and strength.
Lee et al. [51]	young mice and old mice	*L. plantarum* HY7715	5 weeks	Inhibition of the sarcopenic process in skeletal muscle.
Bindels et al. [52]	mouse model of leukemia	*L. reuteri* 100–23 and *L. gasseri* 311,476	/	Reduction in the expression of atrophy markers in muscles.
Varian et al. [53]	*Apc^Min/+^* mice and *wildtype* littermates for experiments involving cancer cachexia	*Lactobacillus reuteri* ATCC-PTA-6475	/	Symbiotic bacteria through FoxN1 and thymic stimulation provide possible alternatives for cachexia prevention.

**Table 4:** Summary of the reviewed studies concerning skin.

Study	Study Population	Probiotic Strain	Duration of the Treatment	Results
Brognara et al. [90] Systematic review	12 in vitro, 8 in vivo studies and 2 human studies	/	/	Preliminary evidence supports the use of specific strains of probiotics in certain clinical settings, such as infected chronic wounds.
Mohsemi et al. [92] Randomised, double-blind, placebo-controlled trial	60 subjects (40–85 years) with grade 3 diabetic foot ulcer	*Lactobacillus acidophilus*, *Lactobacillus casei*, *Lactobacillus Fermentum* and *Bifidobacterium bifidum* (2 × 10^9^ CFU/g each)	12 weeks	Probiotic supplementation led to significant reductions in ulcer length, width, and depth.
Peral et al. [94]	80 burned patients	*L. plantarum*	/	In second-degree burns, *L. plantarum* was as effective as the SD-Ag one. In third-degree burns, *L. plantarum* was more effective.

**Table 5:** Summary of the reviewed studies concerning SAP side effects.

Study	Study Population	Probiotic Strain	Duration of the Treatment	Results
Shen et al. [99] Systematic reviews	19 studies	/	/	Administration of probiotics closer to the first dose of antibiotic reduces the risk of CDI by >50% in hospitalised adults.
Kaku et al. [102] Placebo-controlled trial	33 patients who underwent spinal surgery	*Enterococcus faecium* 129 BIO 3B-R	5 days	Streptococcus gallolyticus and Roseburia were significantly decreased in the probiotics group.
Lau et al. [100] Systematic review and meta-analysis	/	/	/	Significant risk reduction in Clostridium difficile-associated diarrhoea in patients receiving antibiotics associated with probiotics.
Nagamine et al. Retrospective case-control study [101]	29 cases and 120 control	*Streptococcus faecalis*, *Bacillus mesentericus*, and *Clostridium butyricum*	more than 14 days	Risk reduction in Clostridium difficile infection.

## Data Availability

Not applicable.
